# Elucidating the Trajectory of the Charge Transfer Mechanism and Recombination Process of Hybrid Perovskite Solar Cells

**DOI:** 10.3390/ma14112698

**Published:** 2021-05-21

**Authors:** Joseph K. Kirui, Solomon Akin Olaleru, Lordwell Jhamba, Daniel Wamwangi, Kittessa Roro, Adam Shnier, Rudolph Erasmus, Bonex Mwakikunga

**Affiliations:** 1 Physics Department, University of Venda, Thohoyandou 0950, South Africa; joseph.kirui@univen.ac.za (J.K.K.); lordwell.jhamba@univen.ac.za (L.J.); 2 CSIR-Energy Centre, Council for Scientific and Industrial Research, P.O. Box 395, Pretoria 0001, South Africa; kroro@csir.co.za; 3 DST/CSIR—National Centre for Nano-Structured Materials, P.O. Box 395, Pretoria 0001, South Africa; bmwakikunga@csir.co.za; 4 Physics Department, Yaba College of Technology, P.M.B 2011, Lagos 100001, Nigeria; 5 School of Chemistry and DSI-NRF Centre of Excellence in Strong Materials (CoE-SM), University of Witwatersrand, Johannesburg 2050, South Africa; Daniel.Wamwangi@wits.ac.za (D.W.); adamshnier@gmail.com (A.S.); Rudolph.Erasmus@wits.ac.za (R.E.); 6 Physics Department, Arcadia Campus, Tshwane University of Technology, P.O. Box 680, Pretoria 0001, South Africa

**Keywords:** interface, recombination, ideality factor, perovskite solar cell

## Abstract

Perovskite-based solar cells (PSCs) have attracted attraction in the photovoltaic community since their inception in 2009. To optimize the performance of hybrid perovskite cells, a primary and crucial strategy is to unravel the dominant charge transport mechanisms and interfacial properties of the contact materials. This study focused on the charge transfer process and interfacial recombination within the n–i–p architecture of solar cell devices. The motivation for this paper was to investigate the impacts of recombination mechanisms that exist within the interface in order to quantify their effects on the cell performance and stability. To achieve our objectives, we firstly provided a rationale for the photoluminescence and UV-Vis measurements on perovskite thin film to allow for disentangling of different recombination pathways. Secondly, we used the ideality factor and impedance spectroscopy measurements to investigate the recombination mechanisms in the device. Our findings suggest that charge loss in PSCs is dependent mainly on the configuration of the cells and layer morphology, and hardly on the material preparation of the perovskite itself. This was deduced from individual analyses of the perovskite film and device, which suggest that major recombination most likely occur at the interface.

## 1. Introduction

Hybrid perovskites are currently regarded as inspiring novel materials for basic studies and practical applications in optoelectronic devices because of their unusual and useful properties emerging from their mix of organic and inorganic constituents [1]. The perovskites are direct bandgap semiconducting materials with strong absorption edges and high luminescence efficiency and without a Stokes shift. Furthermore, perovskites demonstrate exceptional optoelectronic properties, such as effective photon recycling, high free-carrier diffusion, and an ambipolar nature to the transfer of electrons and holes [2,3,4]. These intrinsic physical properties are among the main parameters responsible for the high PCE values in PSCs, and are also the reason why perovskite materials can function in various configurations. Regardless of the fact that the present record for the highest PCE value in PSCs is 25.2% [5], a profound knowledge of the processes regarding charge transport and recombination among layers and within the perovskite layer is a primary necessity for more improvements [3]. Additionally, the interfaces between the perovskite film and the contact layers are the main determinants for the PCE and have little impact on the stability of HPSCs [5].

Thin-film perovskite solar cells have a sandwich architecture; during the photovoltaic process, the incident photons generate electrons and holes when they are absorbed by the perovskite material. These free carriers move within the perovskite to the interface of individual charge specific contacts, and thereafter are collected in electrodes. In a simplified way, electrons should be moved from the absorber film to electrodes without losses. Subject to the deposition strategies [3,6] and the composite of the perovskite [7] being employed, energetic disorder can occur in the crystal boundaries, which serve as recombination points for losses. As a means to limit these recombination locations, tremendous attempts have been made to enhance the properties of the perovskite materials during compositional engineering [7], interface modification [8], or processing techniques such the gas-phase method [9]. Nevertheless, interfaces are susceptible to losses owing to energy level misalignment, poor contacts, or defects at interface, which result in high charge accumulation, leading to a decline in the efficiency of the solar cell, particularly in open-circuit state [10,11]. Specifically, it remains unclear how the observed alterations directly affect the transfer of charge carriers throughout the interface.

Due to the multilayered structure of perovskite solar cells, interfaces in the heterostructure play a key role in the separation of charges and also influence the long-term stability of the device. Hence, it is necessary to design and regulate the charge extraction throughout the contacts so as to limit the loss of energy, increase the quality of the device, and also strengthen the stability. The presence of different contact materials complicates the formation of ohmic contacts as a result of differences in bandgaps between materials, which results in a Schottky interface.

Radiative recombination of charge carriers appears not to contribute significantly compared to the nonradiative recombination process in the PSCs [12,13]. The nonradiative recombination occurs when the trapped charge carrier, which is positioned at the energy level across the bandgap, recombines with a carrier of opposite charge. However, the traps may be basically built-up either at the grain boundaries of the perovskite material [14] or at the interfaces between the perovskite and contact layers [15]. It appears that the two kinds of accumulation occur in the PSCs and affect the overall performance [12,13]. Overall, it has been generally established that interface recombination does influence the properties of the solar cells [12,16]. However, for PSCs, only recently have studies been published on the significance of this kind of recombination [12,17,18,19].

With the aim of unlocking the potential of PCEs for single-junction and tandem perovskite solar cells, it is vital to advance the knowledge of the underlying recombination loss mechanisms. It has been clearly established that nonradiative recombination losses are the key factors holding back the PSCs from reaching their full thermodynamic potential [20,21]. Nonradiative recombination losses reduce both the open-circuit voltage (*V*_oc_) of the device and the fill factor due to the ideality factor being greater than one [20,22].

The origin of nonradiative recombination losses in PSCs continues to be an intensely discussed subject. Initially, a concerted effort was focused on limiting trap-assisted recombination at defects in the perovskite bulk or at grain boundaries [20,23,24]. Indeed, appreciable enhancements have been accomplished, resulting from the improvements in the fabrication and design of perovskite to enhance the grain size and improve the crystallinity and material optimization. However, issues regarding the interfacial properties of contact materials and their many impacts on the performance and stability of such devices need to be resolved to allow full commercialization of PSCs.

Most of the previous studies on the electrical characterization of perovskite solar cells focused on mesoporous structured cells [25,26]. Because of the similarity in the mesoporous perovskite device structure with that of dye-sensitized solar cells (DSSCs), early perovskite studies sought to interpret the device response in a similar manner. The studies centered around the challenges in resolving the impacts of the mesoporous layer from the intrinsic behavior of the perovskite active layer. The primary reason behind the emphasis on mesoporous cells was that it appeared to be easier to make high-performance cells with good steady-state properties (such as low hysteresis), utilizing the mesoporous as opposed to the planar heterojunction structure [27]. However, to investigate the electronic properties of the perovskite layer itself effectively, it is advisable to study planar heterojunction devices [10]. In this type of FTO–ZnO–perovskite–Spiro-OMeTAD–Au device, the flat perovskite layer is in contact with two different carrier transport materials, thus making it easier to examine the intrinsic behavior. In addition, all of the materials used in this device are dopant-free. Our aim here is two-fold: firstly, to investigate the charge transfer process without the added complexity of dopant materials, and secondly to assess the impacts of recombination mechanisms that exist within the interface, together with the effects on the stability of the device.

To the best of our knowledge, apart from previous articles on devices based on the mesoporous architecture [25,26], no studies of the planar architecture of dopant-free PSCs have been reported so far. The focus of this paper is on the studies of the basic photovoltaic parameters of PSCs and their optoelectronic properties. In the first part, we account for the photoluminescence and UV-Vis measurements on perovskite thin film to elucidate different recombination pathways. In the second part, we use ideality factor measurements (I–V curve) and impedance spectroscopy to establish the recombination mechanisms in the device.

## 2. Materials and Methods

Our hybrid perovskite was made from organic and inorganic sources using a solution-processed method. The organic source was methylammonium iodide (CH_3_NH_3_I) and the inorganic source is lead iodide (PbI_2_). Most of the materials used as shown in Table 1 were purchased from Sigma Aldrich.

### 2.1. Fabrication of Perovskite Solar Cell

**Figure 1:**
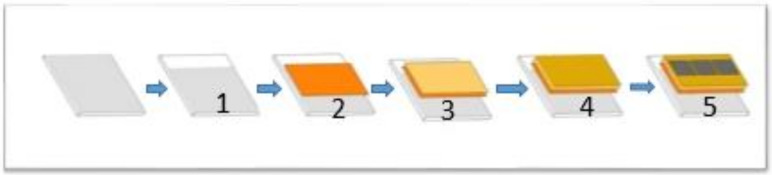
Planar perovskite solar cell fabrication procedures: (**1**) FTO etching; (**2**) ZnO deposition; (**3**) PbI_2_ + MAI + DMSO in DMA deposition; (**4**) HTL deposition; (**5**) gold back contact deposition.

Devices were manufactured on fluorine-doped tin oxide (FTO) coated on glass substrates, as shown in Figure 1. The FTO layer was etched using zinc powder and diluted hydrochloric acid (2M HCL) mixture to enable fabrication of top electrode contact pads without shorting of the devices. The glass substrates were rinsed with water. All etched substrates were cleaned with deionized water and acetone via sonication for about 30 min before rinsing with ethanol and deionized water. Substrates were then dried with N_2_ gas. On this partially etched substrate, zinc oxide was deposited by radio frequency (RF) magnetron sputtering over a ZnO target. The etched FTO-coated substrate was mounted on the rotating stage inside the sputtering chamber and the working gas pressure was kept at 10 mTorr during the sputtering process. High-energy Ar ions bombarded the ZnO target, thereby lifting the target molecules that were deposited onto the FTO-coated glass substrate and creating the ZnO film. The preparation of precursor solutions and spin-coating were carried out under normal room temperature conditions with the presence of oxygen and moisture.

Light absorbing perovskite was deposited on top of the ZnO layer using a single coating procedure accompanied by immediate annealing. Methylamine iodide (CH_3_NH_3_I) (0.160 g) and lead iodide (PbI_2_) (0.460 g) were dissolved in dimethyl sulfoxide (DMSO) and dimethylformamide (DMF) at the stoichiometric ratio of 1:4 (DMSO: DMF). The precursor solution was heated on a hotplate for about 20 min at 70 °C along with stirring for the total reaction. CH_3_NH_3_I and PbI_2_ at a stoichiometric ratio of 1:1 were spread on the substrate through a spin coater. In this spin coating procedure, 0.1 m L of solution was spun at 2500 rpm for 30 s, while 0.2 mL ethyl acetate was spun over the film at 2500 rpm for 10 s after the spin coating commenced. The film was then annealed at 70 °C on a hotplate for about 10 min to enable crystallization of the perovskite layer and subsequently cooled to normal temperature. The features and morphology of the perovskite layer depended on the mixture of the precursor, the speed at which the substrate was spun, and the annealing process requirements, such as the annealing time and temperature used for crystallization. The next step was the deposition via spin coating of the (100 µL) hole transport layer (HTL) with Spiro-OMeTAD at 3500 rpm for 20 s. To prepare the HTM solution in chlorobenzene, 72.3 mg of Spiro-OMeTAD was mixed with 1 mL of chlorobenzene. This provided a simplified approach for preparing HTM without using the hydrophilic lithium salt (Li-TFSI). Finally, gold (Au) was then deposited through a mask as the top electrode in a high vacuum chamber using thermal evaporation. The active area of the cell was estimated to be 0.04 cm^2^.

### 2.2. Thin Film Characterization

The X-ray diffraction (XRD) measurement was clearly confirmed using a XPert Pro X-ray diffractometer from Panalytical Ltd. (Eindhoven, Netherlands) with Cu-K_α_ radiation. Optical absorption profiles were performed using a model lambda 750 Perkin Elmer UV–visible spectrophotometer with wavelengths between 200 and 1400 nm. The photoluminescence (PL) spectrum was measured using a Fluorolog-3 spectrophotometer with an excitation wavelength of 514.5 nm at normal temperature. An Auriga scanning electron microscope from Zeiss (Berlin, Germany) was used to examine the surface morphology of the film. A Veeco Multimode atomic force microscope (AFM) was used to establish the thickness and roughness of the film.

### 2.3. Device Characterization

Current versus voltage (I–V) measurements of the device were performed under AM 1.5G illumination at 1000 Wm^−2^ using a solar simulator equipped with a source meter.

Electrochemical impedance spectroscopy (EIS) measurements using a Bio-LogicVMP3 potentiostat–galvanostat, Seyssinet-Pariset, France were performed under 1 sun illumination with different DC bias voltages ranging from −5.0 to 5.0 V. A small AC voltage perturbation of 5 mV was applied during the measurement at frequencies ranging from 100 mHz to 1 MHz. The spectra were fitted using Z-View software, Seyssinet-Pariset, France.

## 3. Results

### 3.1. Structural Features and Grain Size

**Figure 2:**
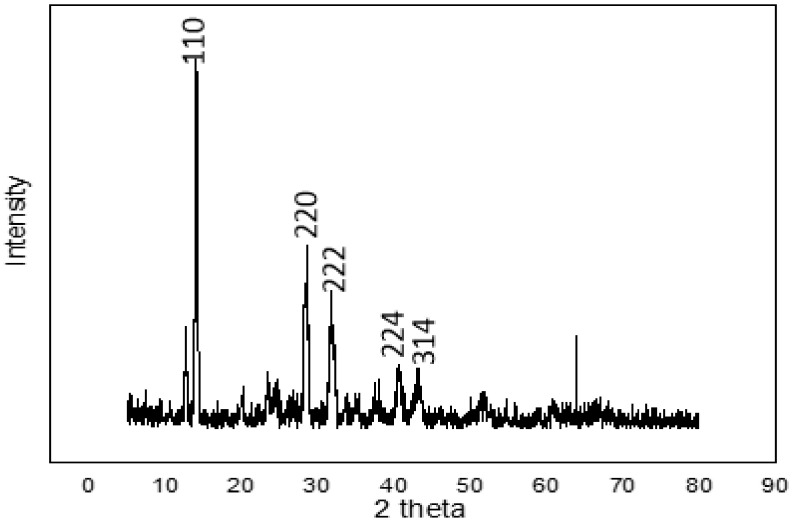
XRD profile of the solution-processed perovskite thin film on FTO glass substrate prepared under ambient conditions.

Figure 2 shows the XRD pattern of the perovskite thin film. The major diffraction peaks situated at 14.1°, 28.4°, 31.9°, 40.6°, and 43.2° for the 2θ scan correspond to the miller indices (110), (220), (222), (224), and (314), respectively. These peaks corroborate those for perovskites obtained by other researchers [28,29,30]. According to the crystal planes produced, we can confirm the formation of perovskite (CH_3_NH_3_PbI_3_), since the peaks that corresponded to lead iodide and methylamine iodide were not observed and the perovskite film was the crystalline perovskite phase. X-ray diffraction affirmed the suitable reaction between PbI_2_ and CH_3_NH_3_I. The perovskite crystallinity and the quantity of PbI_2_ residue are essential to the performance of perovskite solar cells. Excess PbI_2_ residue would hinder injection of electrons from the perovskite to the ZnO layer, thus limiting the performance of the PSCs.

**Figure 3:**
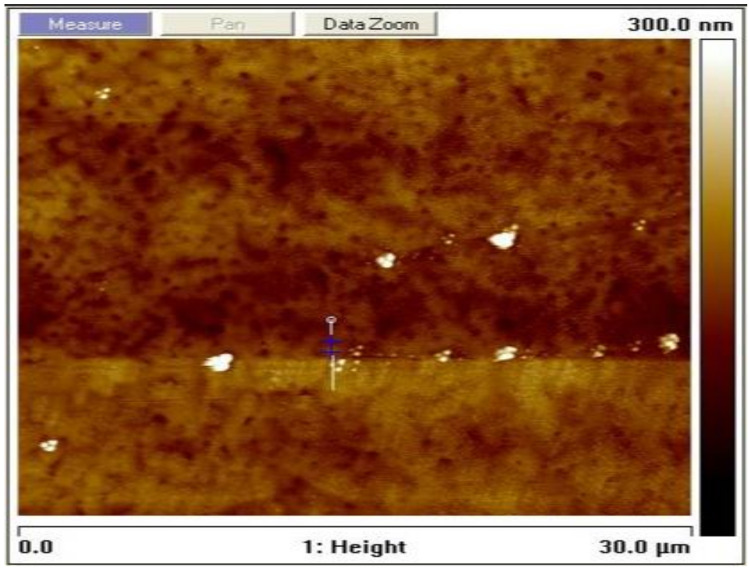
Topographical image of the solution-processed perovskite thin film prepared under ambient conditions.

**Figure 4:**
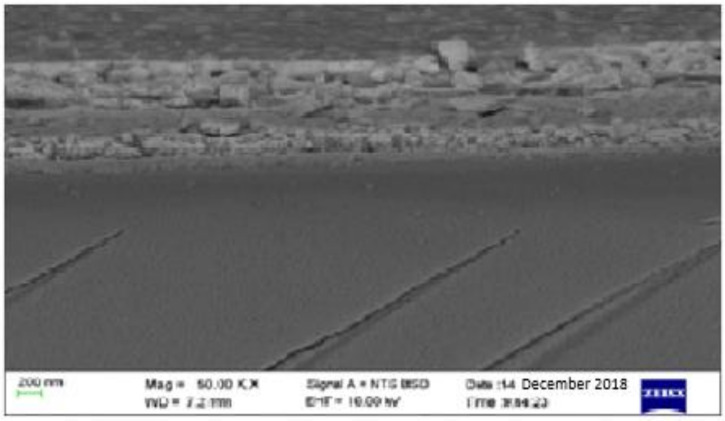
SEM micrograph for the solution-processed perovskite thin film prepared under ambient conditions.

The grain size was estimated to be 30 nm according to the Scherrer formula using the calculation technique used in [31,32]. The surface topography of the film as measured using an AFM at a resolution of 30 × 30 µm^2^ to yield an rms roughness of 7.7 nm is shown in Figure 3. The average height-to-height correlations correspond to the morphological image in Figure 4 for the perovskite film prepared by employing DMSO additive in the solvent. It is, therefore, obvious that the use of ethyl acetate as an antisolvent leads to homogeneous crystallization of the perovskites with minimal pinholes.

### 3.2. Photophysics of Perovskite Polycrystalline Thin Film

The working principles of PSC are strongly dependent on the quality of the perovskite film. Hence, it is very imperative to investigate the luminescence properties of the film before layer coupling so as to identify the causes and locations of recombination loss within the device. It must also be noted that the best quality film and spectroscopic factual knowledge of intrinsic properties are vital for the development of modern perovskite applications, since basic research usually forms a pillar of technological advancement.

#### 3.2.1. Transmission and Emission Spectra

**Figure 5:**
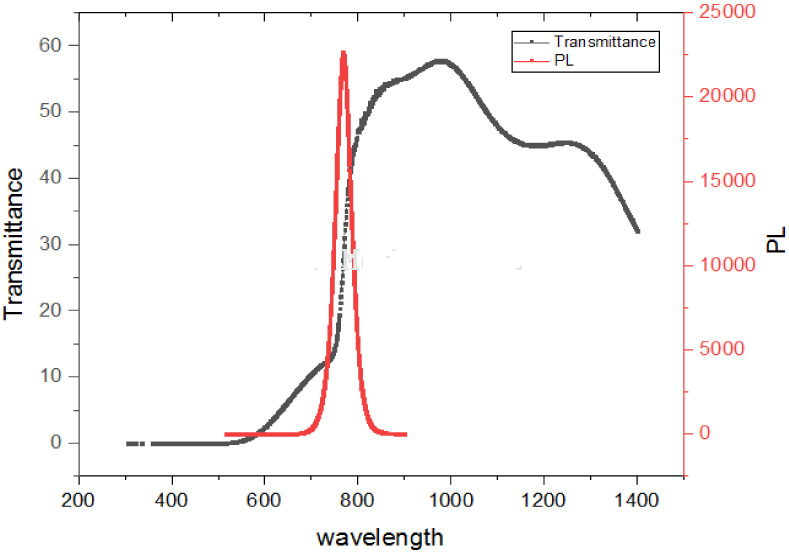
Transmission and PL properties of solution-processed perovskite thin film.

The absorption edge in our hybrid perovskite thin film was located at around 797.50 nm and corresponded to a bandgap of 1.55 eV. The perovskite layer with a thickness of 300 nm showed that the absorption was in the red and near-IR regions, thus suggesting a potential match to the highest photon flux in the solar emission spectrum. The absorption edge of CH_3_NH_3_PbI_3_ displayed in Figure 5 is evidence of the low defect density and step-like absorption, which is necessary for improving solar cells, as stipulated according to the detailed balance theory [33,34]. Furthermore, the absorption here exceeds 700 nm and is a beneficial quality for optoelectronic devices.

There is an absorption edge around 600 nm that is not as sharp as the one around 800 nm. This implies that the absorption range of lead halide perovskite is not confined to the visible region, as reported in some studies [61]. However, by further enhancing the morphology and composition of perovskite materials, the near-infrared (NIR) photo responses of PSCs can be optimized.

Photoluminescence (PL) provides information on the degree of crystallinity, quantum efficiency, electronic properties, existence of traps and defect states, recombination process, and phase transformation in materials. This information may be derived from the linewidth, intensity, and content of the PL spectrum, as displayed in Figure 5.

An important measure for defining the optical feature of a material using the PL spectrum is the width of the emission band, as displayed in Figure 5. When the full-width at half-maximum (FWHM) of the PL shows smaller values (789.99 nm–748.16 nm), this is an indication of excellent structural uniformity, such a width of several quantum wells and formation uniformity. The excitation of the thin film using the 514.5 nm Ar^+^ line showed a main intense peak located at about 769.79 nm corresponding to band-to-band emission at air temperature. The film with smaller crystallites could reveal better luminescence intensity than for bigger crystallites. This is consistent with the crystallite size obtained from the Scherrer formula. There is no evidence of the quantum confinement effect, since the crystalline domains (as indicated by SEM and XRD) are larger than the recorded Bohr radius (2.2 nm for CH_3_NH_3_PbI_3_) [35]. There are two conclusions drawn from the spectrum. First, the sharp peak intensity is an indication of a radiative recombination mechanism (Wannier–Mott excitons). Secondly, the energy of the PL emission (transition energy) is red-shifted by 0.16 eV, which is desirable due to the emission (absorption) at longer wavelengths. The observation of the emission band being red-shifted from the absorption edge is commonly attributed to defect states [36], such as structural defects, vacancies, and impurities. Generally, a red-shifted emission peak is as a result of spontaneous radiative recombination inside such trapped states as compared to the shift from the band edge transition, while the passivation procedure can blue shift the PL peak.

#### 3.2.2. Absorption Coefficient (α)

The absorption coefficient of a material at a particular electromagnetic wave describes energy transfer through carrier excitation. It outlines how a material light of a particular electromagnetic wave can enter before it is absorbed. The absorption coefficient (*α*) can be calculated using the Beer–Lambert relation in the equation *α* = 2.303 × *A/t*, where *A* is the absorbance and *t* represents the thickness of the thin film.

The absorption is equal to the flow of the charge carriers from the valence to the conduction band, which is used to evaluate the bandgap of the material. In accordance with the absorption, the value of the absorption coefficient (1.84 × 10^4^ cm^−1^) was evaluated, similarly to other studies [62]. The absorption of the MAPbI_3_ film exhibits a strong edge around the wavelength of 797.50 nm, in close agreement with c-Si, GaAs, CIGS, and CdTe [37]; thus, very thin films are suitable for PSCs because of the long exciton diffusion lengths, in contrast to the organic semiconductor, which has short exciton diffusion lengths.

#### 3.2.3. Anti-Stokes Shift

The unusual occurrence of anti-Stokes shift luminescence, in which photons are emitted with greater energy than absorbed, is clearly shown in Figure 5. The above behavioral pattern seen in Figure 5 affirms that CH_3_NH_3_PbI_3_ has almost no defects and the free carriers in the bands regulate the optical responses. It is also suggested that excitons do not seem to be paramount for CH_3_NH_3_PbI_3_, which is in agreement with the absorption spectrum displayed in Figure 5. A potential reason for this rare behavioral pattern is that it is the result of homogeneous broadening [38]. A suggestion was made that an effective electron–phonon interplay is responsible for the high anti-stokes PL [39].

In summary, the peculiar anti-Stokes shift suggests a strong electron–phonon coupling in MAPbI_3_, which may be useful for particular applications, such as up-conversion of nanomaterials and bio-imaging. Our results suggest that optical excitation in perovskite is generated by Wannier–Mott excitation [40], which indicates the creation of free charge carriers responsible for the photovoltaic effect.

### 3.3. Photovoltaic Performance Measurement

The performance of the whole device likewise depends on the interfacial contacts with the charge-extracting materials, following the idea that “the interface is the device”.

Measurement of the light intensity dependence of the current–voltage (I–V) was demonstrated to be an effective instrument for pinpointing the prime recombination loss processes in organic photovoltaic (OPV) devices [41,42,43], which is also applicable to perovskite solar cells.

**Figure 6:**
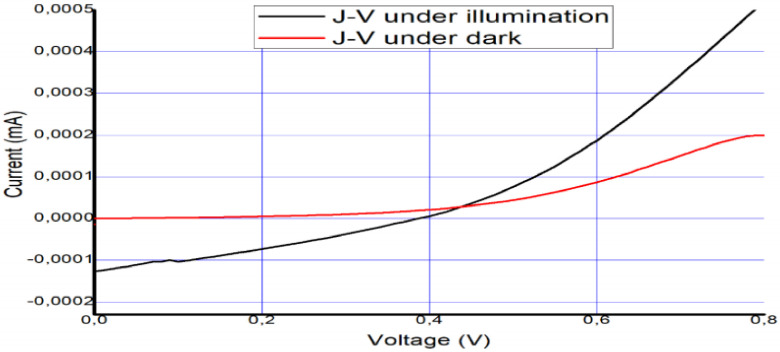
Measured I–V curve of MAPbI_3_ under AM 1.5 and under dark conditions on 12 December 2018.

**Figure 7:**
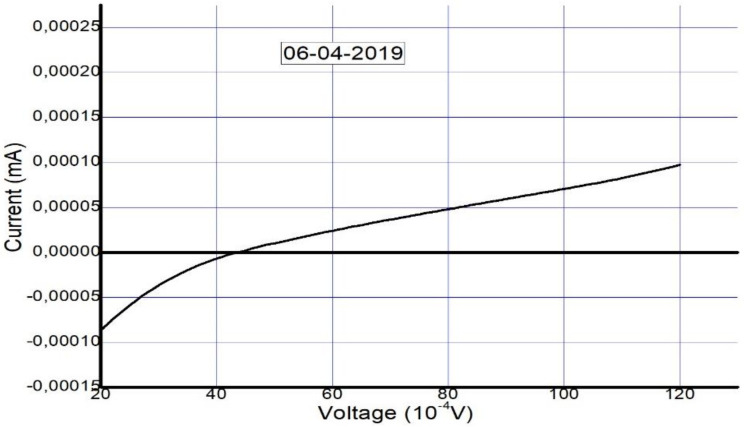
Measured I–V curve of MAPbI_3_ after five months (on 6 April 2019).

The solar cell was fabricated and tested on 12 December 2018. To confirm its long-term stability, five months later (6 April 2019), the device was tested again to check its performance and level of stability. The photovoltaic parameters that were collected from the I–V curves in Figure 6 and Figure 7 are summarized in Table 2 and Table 3.

The cell was fabricated under ambient conditions without a glovebox. It was kept under normal conditions without encapsulation. Our aim was to study charge transport mechanisms of the device without using dopants, a glovebox, or encapsulation. This would enable us to gain a better understanding of the working mechanism of the cell.

The performance of the PSCs can be determined from the short circuit current density, the open circuit voltage, and the fill factor (FF). Generally, the FF is highly dependent on the ratio of the magnitudes of series resistance (*R_S_*) and shunt resistance (*R_sh_*).

**Figure 8:**
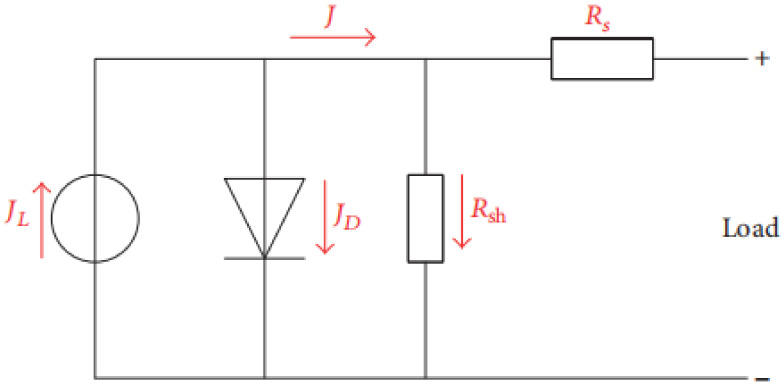
Equivalent circuit of the perovskite solar cell.

The equivalent circuit of the PSC is displayed in Figure 8. The output current density *J* can be stated by Equation (1):

(1) $$J=J_{0}(e^{\frac{q\left(V-JR_{s}\right)}{nKT}}-1)+\frac{V-JR_{s}}{R_{sh}}+J_{\mathrm{sc}}$$

where *J*_0_ typifies the reverse saturation current density, *n* is the ideality factor, *K* is the Boltzmann’s constant, *T* is the temperature, and *q* is the electron charge.

The I–V curve after 5 months suggests the presence of a Schottky barrier due to the increase in series resistance. This occurred as a result of the decomposition of the perovskite layer and degradation of the interfacial carrier transport layer, similar to the results in [63]. We take this as evidence of degradation that occurred due to unencapsulation of the device over a long period.

It was observed that the series resistance *R_s_* conveys the entire conductivity of the device, which is strongly correlated with its internal carrier mobility, while the shunt resistance *R_sh_* applies to the loss of photogenerated charges during carrier recombination inside the device, especially at layer interfaces.

The high *R_s_* value implies that the interphase contact was small with high recombination and low hole mobility in the HTM, which means a low photocurrent would be produced.

*R_sh_* is intimately connected with the charge recombination at interfaces within solar cells. A lesser charge recombination results from a higher *R_sh_*, and vice versa. The low shunt resistance suggests that the power loss in the solar cell across the alternate current path was very high, leading to low FF and electron mobility.

To further evaluate the performance of our hybrid PSC, the following photovoltaic parameters were determined. The incident photon to current conversion efficiency (IPCE) was calculated using the relation IPCE% = 1240 (*J_sc_*/Pin𝜆). Here, an IPCE of 52.8% was obtained, which suggests that the ZnO–perovskite heterojunction utilizes the advantages of each component. This includes the improved light absorption of CH_3_NH_3_PbI_3_ and fast electron transfer of ZnO. Hence, the size of the IPCE, also known as the external quantum efficiency (EQE), relies on the level of light absorption by the solar cell and how much of the absorbed light is transformed to charge carriers, which are extracted. This is why the IPEC is usually affected by reflection and external loss of the photons.

The internal quantum efficiency (IQE), also known as the absorbed photon to current conversion efficiency (APCE), serves as a measure of the inherent performance of a device, including its charge separation and charge injection efficiency. The IQE is usually higher than the EQE. A small IQE shows that the active layer of the solar cell cannot effectively utilize the photons. The equation for the APCE is given as follows: APCE = EQE/1 − reflection.

Our solar cell showed better stability and great light harvesting and charge separation efficiency, although the conversion efficiency was reduced due to the thickness of the perovskite layer, as well as certain other factors that will be discussed in the next section. The values of *V_o__c_* and *J_sc_* obtained could be related to the low hole mobility in the hole transport material due to the absence of lithium salt and interfacial recombination loss, resulting in low efficiency.

### 3.4. Impedance Response of Planar Perovskite Solar Cell

To gain insight into the separation of light-induced charges and recombination at the interfaces, we conducted EIS measurements and ideality factor measurements on the device.

**Figure 9:**
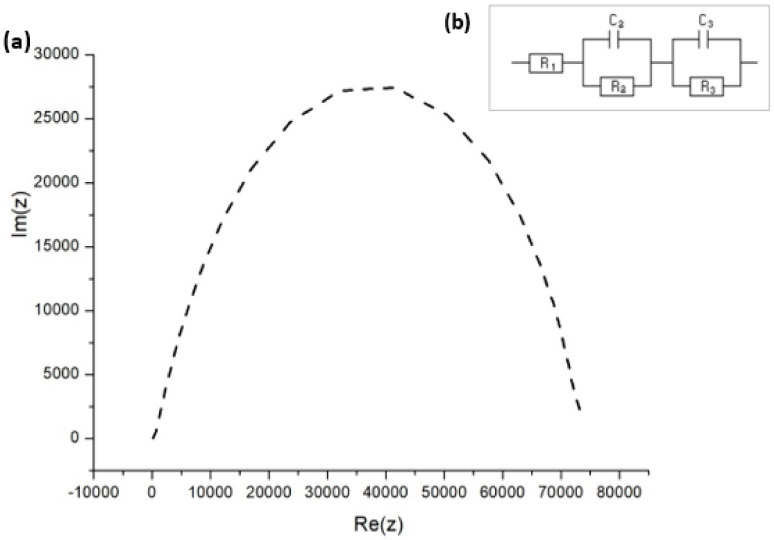
(**a**) Nyquist impedance spectrum for FTO–ZnO–perovskite–Spiro-OMeTAD–Au device. (**b**) EIS equivalent circuit employed to fit the impedance response.

The dynamic response of any solar cell is determined by various fundamental processes, including charge transport, charge storage, electron hole recombination, and interfacial charge transfer. Each of these processes can be reflected in the impedance response. The EIS measurements address charge extraction, which controls short-circuit conditions and charge recombination, which in turn determine the open-circuit conditions, allowing for complete investigation of the solar cell. From the Nyquist plot in Figure 9a, the high and low frequency impedance characteristics were observed while testing the FTO–ZnO–perovskite–Spiro-OMeTAD–Au device.

An equivalent circuit in Figure 9b was employed to fit the data in the Nyquist plots, the respective fitting parameters for which are provided in Table 4. *R*_1_ is the series resistance of the cell, *C*_3_ * = Cµ* is the chemical capacitance of the cell, and *R*_3_ is the recombination resistance. *C*_2_ and *R*_2_ are respectively the capacitance and resistance of hole transport materials [44,45]. The equivalent circuit model that we adopted has been used extensively to explain the EIS data of PSCs made using the solution process [46,47].

In our experiments (Figure 9a), the diffusion (that is, Warburg diffusion) of the charge carriers was not observed, which was indicative of two separate impedance indicators: the recombination resistance *R*_rec_ and the trap-dominated chemical capacitance *C_µ_*. There was no information at all with respect to electron transport. There are two possibilities why the Warburg part was absent: the first one is that the conductivity was extremely high and transport resistance became drastically low, while the second possibility is that the presence of the electron transport layer (ETL) network attributed to the very thin ZnO film [48]. Carriers generated in thin layer do not have to diffuse for long before they can be collected at the junction. Therefore, very thin layers would lead to the loss of long-wavelength photon absorption, reducing the photocurrent. The lack of the transport resistance feature in Figure 9a is thought to account for the high diffusion rates and mobilities described for charges in hybrid perovskite materials [49,50].

The capacitive components are expressly shown in the established illustration as being related to the dielectric constant of perovskite and to the surface charge build-up at the interface. The *C*_2_ predominates the capacitive feedback in the high-frequency section of the spectrum and relates to the separation of charges between the perovskite layer and neighboring interfacial materials, while *C*_3_ responds to the well-known low-frequency process that controls the solar cell operation.

Regarding the operating mechanism for the perovskite solar cells on the basis of EIS, the impedance indicators, such as the time constants, resistances, and capacitances, were analyzed. Table 5 shows the impedance parameters to further explain the dynamic processes within the device. Ideally, one can calculate two isolated time constants as the products *τ_H_* = *R*_2_*C*_2_ and *τ_L_* = *R*_3_*C*_3_. These time constants are related to the high- and low-frequency arcs, as long as *C*_3_ *>> C*_2_, which applied in our case.

The shorter time constant *τ_H_* keeps the trend marked by *R*_2_. Considering *C*_2_ is uncoupled from the resistive element *R*_2_, the respective time constant cannot be considered as a characteristic time of any physical mechanism. Conversely, the longer time constant *τ_L_* shows light-independent values during both short- and open-circuit conditions. The light-independent behavior and the same level of magnitude of these time constants are clear signals of the coupling between *R*_3_ and *C*_3_ in a usual kinetic process. In addition to this, *τ_H_* cannot be appropriately regarded as a carrier lifetime due to the fact that *C*_2_ is not a chemical capacitance [51]. At the same time, the electron time constant, *τ_L_*, is attributed to the electrons at the interface. Based on the results, the resistive elements follow the inverse relationship pattern with the light intensity, with the low-frequency resistance *R*_3_ exhibiting a bigger value than the high-frequency contribution *R*_2_. Similarly, capacitive elements also confirm the inverse variation with light intensity. Hence, we argue that resistive components, as well as cumulation capacitance, behave in the reverse manner with irradiation intensity and bring us to the conclusion that both originate from the usual process.

### 3.5. Determination of the Basic Transport Coefficients/Parameters

Electron or hole transport is determined by a gradient of the Fermi level and the transport in the semiconductor, hence creating a loss of free energy of the carriers. Moreover, the collection of carriers to create a photocurrent in the external circuit is competing with recombination mechanisms. Therefore, it is vital to assess and measure the energy losses related to the diffusion length and carrier transport; to this effect, the following fundamental transport coefficients of the electronic carriers, such as the mobility and the diffusion coefficient, *D_n_*, were established in this study. In addition, the transport resistance, *r_t_*, is the main parameter that explains the transport features in EIS. A narrative approach to obtain *D_n_* from *r_t_* is presented below.

The electron diffusion coefficient (*D_n_*) can be represented by the following equation: *Dn* = d^2^/235t_H_. The time constants determined can, thus, be employed to calculate the electron diffusion length (*Ln*), which is given by Ln = (D_n_t_s_)^1/2^. The conductivity can be given in the form of the chemical diffusion coefficient and the chemical capacitance as σn = *C_µ_D*_n_.

Mobility (*µ*) is the basic quantity that decides the diffusion length, given as *L* = √*D𝜏*, where *D* is the diffusion coefficient, given as: *D* = *µq*/*k*_B_*T, q* is the elementary charge, *k*_B_ is the Boltzmann constant, and *T* is the temperature.

We show that the electron diffusion length is greater than 1 µm, which is one of the most reported values for 3D perovskite polycrystalline films [52] and much longer than that of the low-dimensional perovskite [53]. This superior carrier diffusion length originated from the enhanced 3D perovskite stability without a dopant. The charge carrier mobility contributes significantly in charge extraction to the electrodes. Delaying charge mobility may enhance the potential for recombination, being accountable for lowering *V_oc_*. This suggests that the mobility plays an important role in managing *V_oc_*_._

### 3.6. Charge Injection Mechanism and Recombination

**Figure 10:**
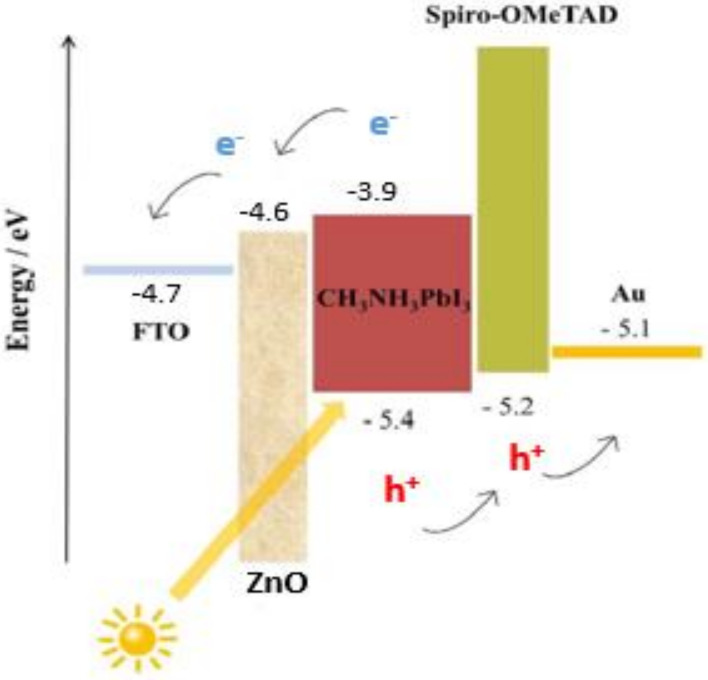
Energy band diagram of the fabricated perovskite solar cell structure, consisting of an FTO electrode, ZnO electron transport layer, light-absorbing MAPbI_3_, spiro-OMeTAD hole transport layer, and gold contact.

In this work, we fabricated the n-i-p device structure. The energy band diagram of the fabricated perovskite solar cell employed in our investigation is depicted in Figure 10. The perovskite layer is the light-absorbing material that generates the charge separation, pushing electrons to the n–i junction and holes to the i–p junction. Our findings from ideality factor and impedance spectroscopy suggest the presence of nonradiative recombination across the bandgap, leading to suppression of *J_sc_*. Furthermore, the suppression of *V_oc_* could be a result of delayed charge mobility at the perovskite–spiro-OmeTAD interface due to the absence of lithium salt in spiro-OmeTAD.

The major junction determinant that governs the charge injection mechanism is the barrier potential, which therefore is always mostly regulated via defects, interface dipoles, Fermi level alignments [54], energetic disorder provided by the roughness at metal–organic semiconductor contacts [55], and defects. In some cases, image force also contributes by generating current backflow [56]. Furthermore, the ideality factor (*n*) is one of the diode I–V characteristic parameters. It is an indicator of charge recombination mechanisms within the semiconductor diode. If the recombination of electrons and holes occurs within the bandgap (i.e., direct recombination or bimolecular recombination), the diode ideality factor is *n* = 1, while the ideality factor of 2 is defined as recombination through defect traps; that is, recombination centers [57]. Diode ideality factors greater than 2 are rare. A possible reason for such high ideality factors is a high rate of interfacial recombination [58,59,60]. The ideality factor influences the fill factor of the solar cell, as an increase in *n* leads to a decrease in the fill factor. This is why the initial approach to the source of the charge recombination that takes place in a device can be considered by checking the ideality factor.

**Figure 11:**
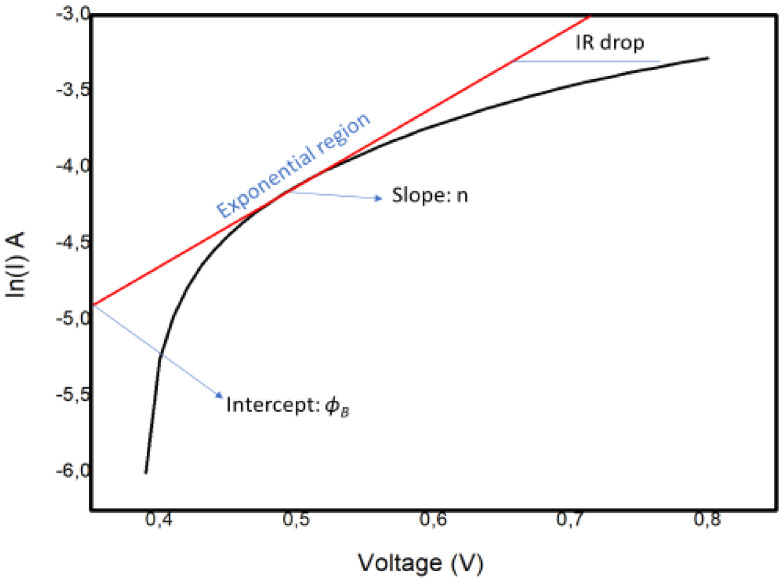
The ln I–V characteristics for the Schottky barrier junction type in the FTO–ZnO–perovskite–Spiro-OmeTAD–Au device.

Additional study is required into the connections between ionic arrangements and recombination. Apparently, interfacial properties control the operation of the device. In light of this, we considered the effects of the interfacial carrier injection process and the recombination mechanism by bringing focus onto the basic interface physics responsible for the observed efficiency of the perovskite solar cell. The usual method used to obtain the barrier height and ideality factor is to plot the ln *I* versus the voltage *V,* as explained below. The ideality factor of the device was calculated using the slope of the linear section (the exponential region of the forward bias of the *ln I (V)* characteristics of the plot shown in Figure 11. The slope, which is equivalent to *dv*/*d (*ln *I)*, was utilized in Equation (2) to establish n:

(2) $$n=\frac{q}{KT}\frac{∆V_{F}}{∆\left(\ln I_{F}\right)}$$

The value of the energy barrier was calculated from the forward bias I–V characteristics using Equation (3), in which A * is the Richardson constant:

(3) $$\phi_{B}=-\frac{KT}{q}\ln\frac{J_{s}}{A*T^{2}}$$

Here, we investigated the potential barrier *ϕ_B_* (also known as the Schottky barrier) and ideality factor (*n*) of the device.

The graph shows a nonideal curve, since a perfect straight line slope was not obtained. The possible explanations for the nature of the curve could be the following:

- The presence of multiple transport mechanisms, such as quantum mechanical tunnelling through the barrier and current leakage across the contact perimeter, which may have altered the linear section of the ln *I–V* plot, hence making it difficult to extract the saturation current and other parameters;
- The high *R_S_* value, which may have increased the magnitude of the ideality factor.

The values of *ϕ_B_* = 0.20 V and *n* = 3.3 were determined from the semilog *I–V* plot in Figure 11. In our case, the interfacial recombination was dominant, since the value was 3.3. This suggests a negative impact on the transport process at the interface, in turn affecting the overall efficiency of the cell. This is consistent with impedance spectroscopy findings indicating that recombination occurred at the interfaces.

## 4. Conclusions

In summary, having demonstrated the impacts of interfacial recombination through ideality factor measurements (I–V curves) and small-perturbation measurement techniques (EIS), the absence of dopants in our (hybrid) perovskite semiconductor increased the longevity of the device and simplified the charge transport mechanism. In our study, the absorption and charge transport process were satisfactorily investigated. However, the final mechanism, which is the extraction of the photogenerated charge at interface, was problematic. In order to successfully suppress the recombination, especially at interface, energy alignment and suppression of nonradiative defect recombination at the interface with a dopant that is not vulnerable to degradation are recommended. Lastly, the use of the planar architecture for investigation affords a clearer picture of the charge transfer mechanism in perovskite solar cells.

## Figures and Tables

**Table 1:** Material used for different layers in fabricated perovskite solar cells.

Layer	Material
Cathode	Fluorine tin oxide (FTO)
Electron transport layer (ETL)	Zinc oxide (ZnO)
Active layer	Perovskite (CH_3_NH_3_PbI_3_)
Hole transport layer (HTL)	Spiro-OMeTAD
Anode	Gold (Au)

**Table 2:** Measured cell characteristics and electrical parameters for Figure 6. Measured cell characteristics and electrical parameters of the device just after fabrication and then 5 months later.

(**a**)							
*V_oc_*	*J_sc_*	*FF*	*IPCE*	*APCE*	*R_s_*	*R_SH_*	*Ƞ*
0.39 V	3.4 mA/cm^2^	33%	52.8%	65.2%	71.4 Ωcm^−2^	160. 4 Ωcm^−2^	4%

**Table 3:** Measured cell characteristics and electrical parameters of the device just after fabrication and then 5 months later.

Date	*J_sc_*	*V_oc_*	*FF*	*Ƞ*
12 December 2018	3.4 mA/cm^2^	0.39 V	33%	4%
6 April 2019	2.3 mA/cm^2^	0.0044 V	25.4%	0.02%

**Table 4:** Measured impedance parameters of the fabricated perovskite solar cell.

R_1_ (Ω)	R_2_ (Ω)	R_3_ (kΩ)	C_2_ (nF)	C_3_ (nF)
63.16	325.1	44.341	3.612	91.39

**Table 5:** Impedance parameters of the fabricated perovskite solar cell.

τ_H_	τ_L_	L_n_	D_n_	µ
1.17 µs	4.05 ms	9.1 µm	32.63 nm^2^/s	0.003 cm^2^/Vs

## Data Availability

The data presented in this study can be obtained from the corresponding author.
